# A Functional Variant in the Stearoyl-CoA Desaturase Gene Promoter Enhances Fatty Acid Desaturation in Pork

**DOI:** 10.1371/journal.pone.0086177

**Published:** 2014-01-20

**Authors:** Joan Estany, Roger Ros-Freixedes, Marc Tor, Ramona N. Pena

**Affiliations:** Departament de Producció Animal, Universitat de Lleida-Agrotecnio Centre, Lleida, Catalonia, Spain; Clermont Université, France

## Abstract

There is growing public concern about reducing saturated fat intake. Stearoyl-CoA desaturase (SCD) is the lipogenic enzyme responsible for the biosynthesis of oleic acid (18∶1) by desaturating stearic acid (18∶0). Here we describe a total of 18 mutations in the promoter and 3′ non-coding region of the pig *SCD* gene and provide evidence that allele T at *AY487830:g.2228T>C* in the promoter region enhances fat desaturation (the ratio 18∶1/18∶0 in muscle increases from 3.78 to 4.43 in opposite homozygotes) without affecting fat content (18∶0+18∶1, intramuscular fat content, and backfat thickness). No mutations that could affect the functionality of the protein were found in the coding region. First, we proved in a purebred Duroc line that the C-T-A haplotype of the 3 single nucleotide polymorphisms (SNPs) (*g.2108C>T; g.2228T>C; g.2281A>G*) of the promoter region was additively associated to enhanced 18∶1/18∶0 both in muscle and subcutaneous fat, but not in liver. We show that this association was consistent over a 10-year period of overlapping generations and, in line with these results, that the C-T-A haplotype displayed greater *SCD* mRNA expression in muscle. The effect of this haplotype was validated both internally, by comparing opposite homozygote siblings, and externally, by using experimental Duroc-based crossbreds. Second, the *g.2281A>G* and the *g.2108C>T* SNPs were excluded as causative mutations using new and previously published data, restricting the causality to *g.2228T>C* SNP, the last source of genetic variation within the haplotype. This mutation is positioned in the core sequence of several putative transcription factor binding sites, so that there are several plausible mechanisms by which allele T enhances 18∶1/18∶0 and, consequently, the proportion of monounsaturated to saturated fat.

## Introduction

**Figure 1:**
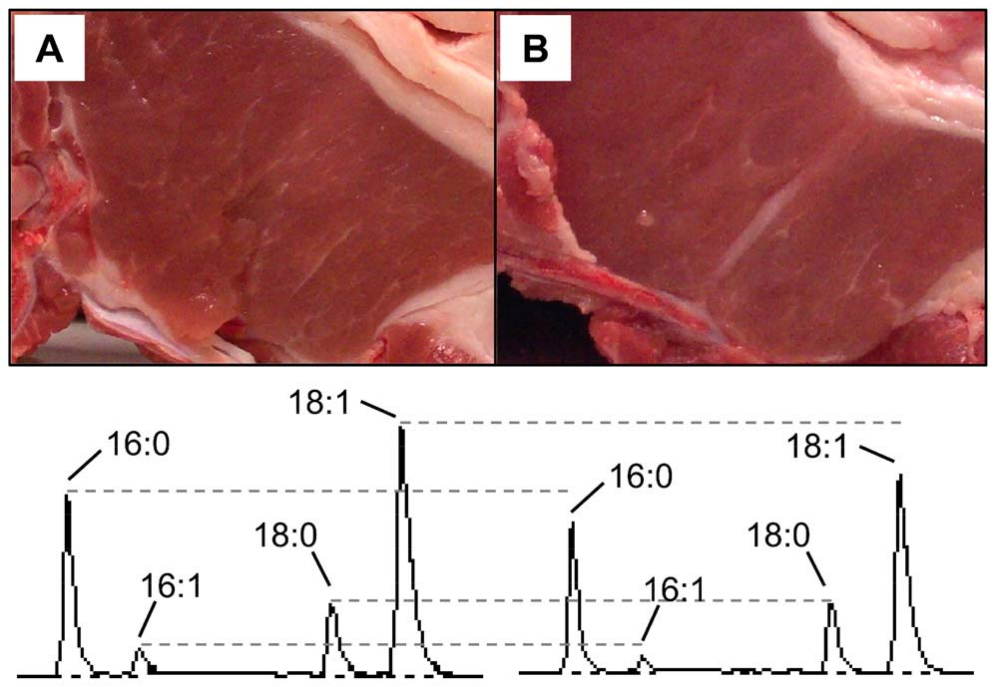
Pork loins with optimal intramuscular fat but different monounsaturated fatty acid content. The monounsaturated pamitoleic (16∶1) and oleic (18∶1) acids are more abundant in the loin in panel A (4.0% and 44.2%, respectively) than in the loin in panel B (3.0% and 41.4%), expressed as percentage with respect to total fatty acids. The peaks of these two fatty acids in the chromatograms below are labelled accordingly, along with those of their respective precursors, palmitic (16∶0) and stearic (18∶0) acids. The desaturation ratios 16∶1/16∶0 and 18∶1/18∶0 are higher in loin A (0.16 and 3.7, respectively) than in loin B (0.12 and 2.8, respectively). Genotyping for *g.2228T>C* in the promoter region of the *SCD* gene revealed that loin A was homozygous for allele T and loin B homozygous for allele C.

Good eating habits are conducive to good health. Total fat and fatty acid content in food affect both human health and food quality and, consequently, they are becoming increasingly important to consumers. There is convincing evidence that a high dietary intake of saturated fat (SFA) increases the risk of lipid metabolism disorders which are common to many human chronic diseases [1]. Conversely, the intake of monounsaturated (MUFA) and polyunsaturated (PUFA) fat has beneficial effects over human health [2]. In this regard, dietary guidelines advice that optimal intake of SFA should account for no more than 10% of the total diet energy, in line with recent findings suggesting that dietary composition may matter for longevity more than calorie count [3]. Worldwide, the demand for meat, but specifically pork, has increased from the 1980s onwards driven by growing human population and incomes [4]. Although pork is rich in bioavailable macro- and micronutrients, it is also a source of dietary SFA [5]. In addition to nutritional aspects, fat content and fatty acid composition also influence relevant manufacturing and organoleptic properties of pork [6], [7]. Thus, high levels of intramuscular fat (IMF) and MUFA are favorably associated to texture, juiciness, flavor, and general acceptability of high-quality products [6], [7] (**Figure 1**). Therefore, a reasonable strategy to deal with both healthy and quality constraints is to substitute dietary SFA with MUFA.

The pork fatty acid composition varies across fat tissues and muscles and it is greatly influenced by the genetic type of the pig, the diet and, in general, by any factor affecting fatness, such as gender or age [8], [9]. In this regard, the use of the Duroc breed is becoming very popular in quality conscious consumer segments because of their high level of IMF relative to subcutaneous fat. However, regardless of the genetic type, the deposition of dietary fatty acids is small compared to fatty acid synthesis, with endogenous oleic (18∶1), palmitic (16∶0), and stearic (18∶0) acids representing more than 80% of the total deposited fatty acids [10]. The stearoyl-CoA desaturase (SCD) is the rate-limiting enzyme required for the biosynthesis of MUFA from SFA. In particular, SCD catalyzes the desaturation of palmitoyl-CoA and stearoyl-CoA substrates at the Δ9 position to produce *de novo* palmitoleoyl-CoA and oleoyl-CoA, respectively. Maintaining a balance in the SCD activity is paramount to optimize health [11], [12] and, therefore, *SCD* expression, both in normal and in disease states, is tightly controlled by dietary and hormonal factors [13]. *SCD* is largely expressed in liver and adipose tissue, responding positively to high carbohydrate diets and negatively to starvation and PUFA rich diets. The ratio of 18∶1 to 18∶0 (18∶1/18∶0) is commonly used as an indirect indicator of SCD activity. Alterations in this desaturation ratio have been linked to cardiovascular disease, obesity, diabetes, and cancer [11]–[15], and correlated with longevity [16]. Recent evidence indicates that SCD also plays an important role in defining plasma and tissue lipid profiles [12].

In pigs, the *SCD* gene is assigned to chromosome SSC14q27 [17]. The position of this gene co-localizes with quantitative trait loci for muscle content of 18∶0 and 18∶1 described in Duroc-based populations [18], [19]. *SCD* is, therefore, an attractive positional candidate gene [20]. In fact, findings so far support that there is genetic variation in the *SCD* gene affecting fatty acid composition of muscle and adipose tissue. Several single nucleotide polymorphisms (SNP) in the *SCD* promoter region have been associated to 18∶0 and 18∶1 content. Yet, results are inconclusive, as either the location of haplotypes is not coincident [21], [22], favorable alleles are swapped [23], or even no association was found [24]. We have been collecting since 2002 samples of subcutaneous fat, muscle, and liver from a full-pedigreed Duroc line [25] and muscle samples from three *ad hoc* pig crossbreds divergent for fatness. Fat content and composition data is currently available for all these samples. Here we use this repository to provide evidence that allele T at SNP *AY487830:g.2228T>C* in the *SCD* gene is a causative mutation that promotes fat desaturation in muscle and subcutaneous fat.

## Results

### Sequence Variation in the *SCD* Gene in Duroc Pigs

The 5′ and 3′ non-coding regions, coding region, and 680 bp upstream on the proximal promoter of the pig *SCD* gene were sequenced in 12 Duroc pigs representing extreme phenotypes for muscle oleic acid content. A total of 18 polymorphisms were identified: three in the promoter and 15 in the 3′ non-coding region (**Table S1**). No variation was found in the sequence corresponding to the *SCD* coding and 5′ non-coding regions.

**Figure 2:**
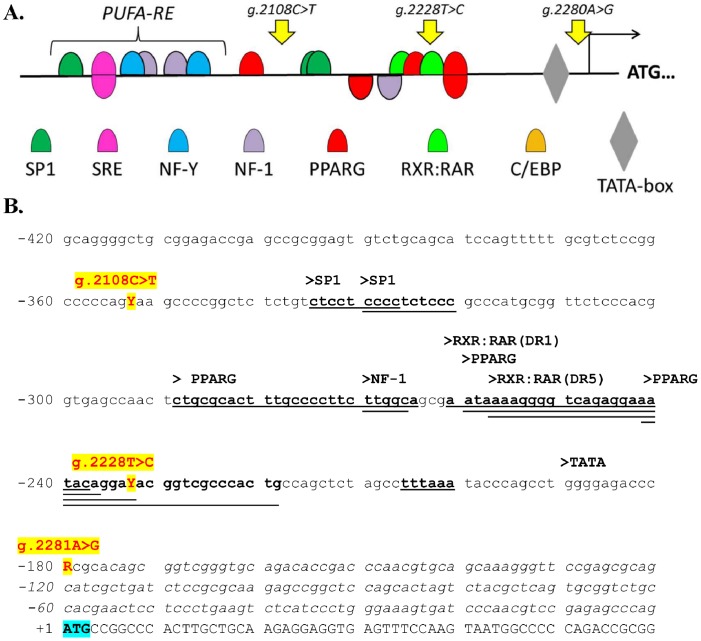
Characterization of the 5′ flanking region to the transcription start site of the pig *SCD* gene. (**A**) Schematic representation of recognition motifs for several transcription factor binding sites in the proximal 5′ flanking region of the pig *SCD* gene. The relative position of the three SNPs polymorphisms identified in this promoter (AY487830: *g.2108C>T, g.2228T>C* and *g.2281A>G*) are indicated. (**B**) Sequence encompassing three SNPs polymorphisms in the promoter region of the pig *SCD* gene. Position numbering is relative to the translation START codon (in blue). The transcription start site is at position −175 (arrow). Coding sequence and the 5′ non-coding region is shown in uppercase and italics, respectively. The motifs for transcription factors SP1, PPARG, NF-1, RAR:RXR and the TATA-box are underlined and notated above the sequence.

The *SCD* transcription unit spans 16,186 bp and includes a coding region of 1,079 bp plus an unusually long 3′UTR of 4,047 bp. Despite being over 12 kb apart, in the Duroc animals analyzed the polymorphisms of promoter and 3′UTR regions formed one haplotype block which displayed >95% overall linkage disequilibrium (r^2^ = 0.965 between SNPs *g.2108C>T* in the promoter and *g.15109A>G* in the 3′UTR). The three SNPs in the promoter region were close together in a 173 bp fragment (**Figure 2**). Given the lack of sequence variation in the coding region of the gene, we focus on the study of the three SNPs in the promoter region as these might potentially influence the *SCD* mRNA expression levels affecting, therefore, the total *SCD* activity of the cells.

### Association of *SCD* Haplotypes with Desaturation Ratios

**Figure 3:**
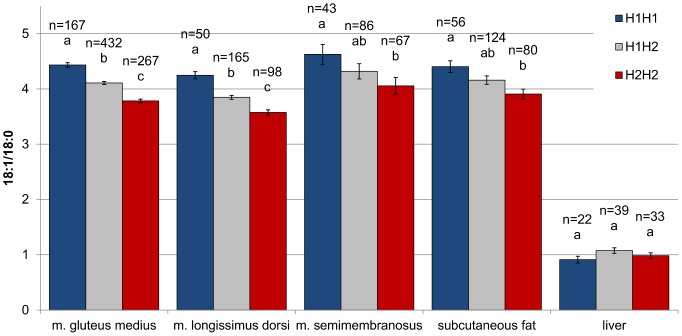
Desaturation ratio by *SCD* diplotype and tissue in purebred Duroc. The presence of haplotype H1 is associated to higher 18∶1/18∶0 ratio both in intramuscular and subcutaneous fat. The H1H1 pigs have a greater 18∶1/18∶0 ratio than the H2H2 animals in the muscles *gluteus medius* (H1H1−H2H2∶0.65), *longissimus dorsi* (H1H1−H2H2∶0.67), and *semimembranosus* (H1H1−H2H2∶0.57), and in the subcutaneous fat (H1H1−H2H2∶0.50), with the heterozygote H1H2 showing an intermediate effect. No difference is observed among diplotypes in liver. Error bars represent standard errors. Columns lacking a common letter within tissue differ (p<0.01, for *gluteus medius* and *longissimus dorsi*; p<0.05, for *semimembranosus* and subcutaneous fat).

**Figure 4:**
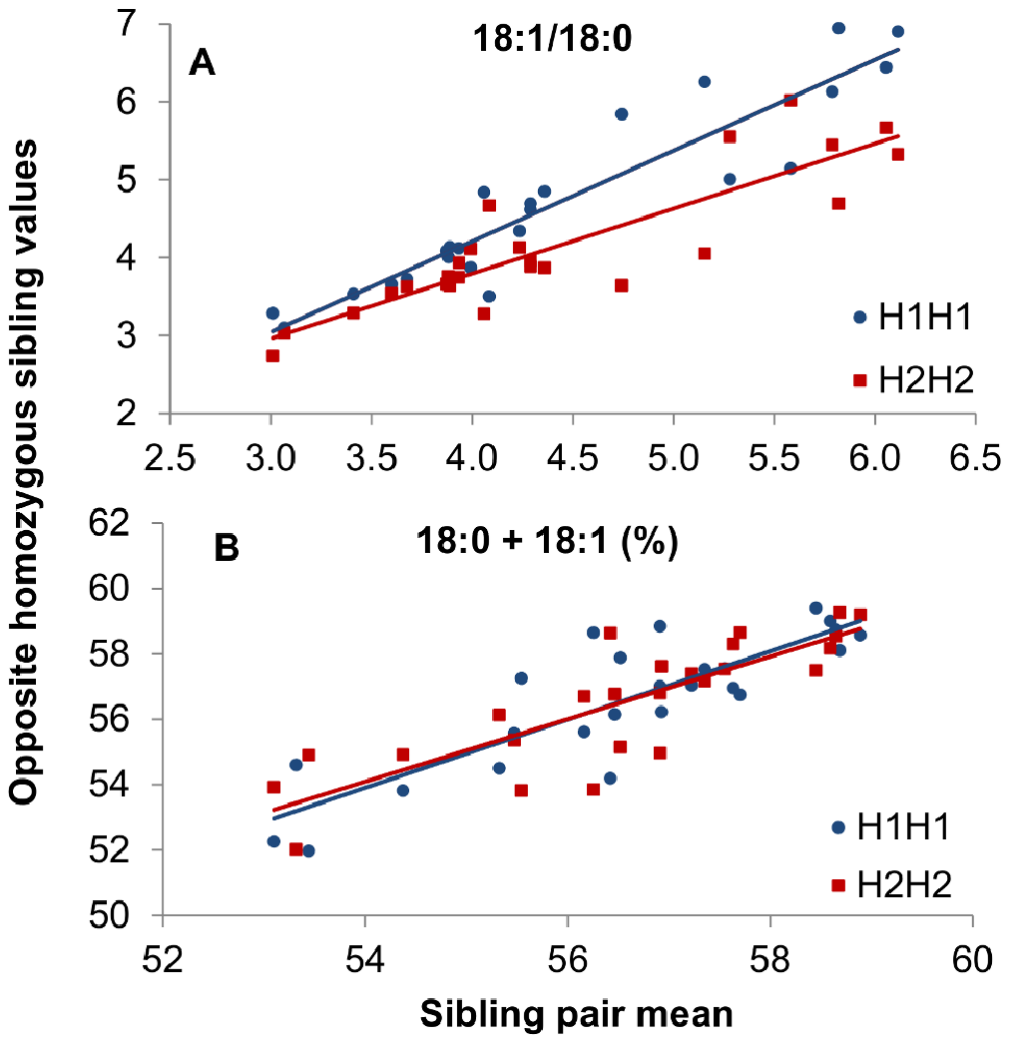
Desaturation ratio in opposite homozygous siblings for *SCD* haplotypes H1 and H2. (**A**) Ratio 18∶1/18∶0, and (**B**) 18∶0+18∶1 content (in percentage of total fatty acids) in the muscle *gluteus medius* of homozygous H1H1 and H2H2 sibling pairs (n = 25) are plotted against the sibling pair mean value. The H1H1 pigs showed a greater desaturation ratio (p<0.01) than their H2H2 sibs but the same 18∶0+18∶1 (p = 0.94) content. The associated p-values were determined using a paired t-test. Regression lines were fitted for each diplotype (blue: H1H1; red: H2H2). The difference between homozygotes for 18∶1/18∶0 increased with 18∶1/18∶0 (p<0.05), with H1H1 sibs showing a trend higher than the expected (1.17±0.10) and H2H2 sibs lower (0.83±0.10). The regression of 18∶0+18∶1 on the litter mean value was not different from the average trend (unity) in both genotypes (p = 0.89).

In a first experiment we genotyped all the available purebred Duroc pigs in the repository (n = 891) which had at least one tissue analyzed for fatty acid composition (Exp 1; **Table 1**). The segregation analysis of the three SNP in this population revealed that they are in strong linkage disequilibrium (r^2^>0.97), with two clearly predominant haplotypes (H1: C-T-A, frequency 43.7%; and H2: T-C-G, frequency 55.5%). The results of the association analysis confirmed that pigs carrying the H1 haplotype had higher 18∶1/18∶0 ratio in the three muscles analyzed (*gluteus medius*, *longissimus dorsi*, and *semimembranosus*) and subcutaneous fat but not in liver (**Figure 3**). We proved that this haplotype behaved additively, with an average additive effect for 18∶1/18∶0 in the muscle *gluteus medius* of 0.33 (**Table 2**), but also that it did not affect the amount of 18∶0+18∶1. Moreover, these effects were consistent across batches, thereby showing both genetic stability over generations and environmental stability against occasional dietary and management changes. A similar trend was found for the 16∶1/16∶0 and the MUFA/SFA ratios (**Table S2**). As a result, the substitution effect of H1 for H2 for MUFA, 18∶1, and 16∶1 in the *gluteus medius* muscle was 1.02%, 0.70%, and 0.30%, respectively. Adjusting these values for the age at slaughter and fat content did not change the results. Because segregation was at intermediate frequencies, the above haplotype variants were able to explain a relevant fraction of the total additive genetic variance for MUFA/SFA (31%), 18∶1/18∶0 (37%), 16∶1/16∶0 (35%), MUFA (20%), C18∶1 (13%), and C16∶1 (25%). However, they did not affect fat content-related traits, including carcass weight, backfat thickness, lean content, and IMF content (**Table S2**), or standard blood lipid indicators (**Table S3**). The favorable effect of H1 on 18∶1/18∶0 was internally validated by comparing opposite homozygote siblings (**Figure 4**). In line with the population-wide results, H1H1 pigs had a greater 18∶1/18∶0 ratio in *gluteus medius* muscle than their corresponding H2H2 sib pairs, with no change in the total content of 18∶0+18∶1.

**Table 1:** Haplotype frequencies of single nucleotide polymorphisms (SNPs) *AY487830:g.2108C>T*, *g.2228T>C*, and *g.2281A>G* at the promoter region of the *SCD* gene in different pig populations.

	Position at AY487830			Genetic type^a^										
				Purebred						Crossbred				
	SNP			Exp 1	Other					Exp 2			Other	
Haplotype	*g.2108C>T*	*g.2228T>C*	*g.2281A>G*	Duroc-1	Duroc-2	Landrace-1	Pietrain	Iberian-1	Wild boar	DU-3×DU-1	IB-2×DU-1	LW-1×L-2	LW-2×L-3	(LW-3×L-4)×DU-4
1	C	T	A	778	26	40	39	81	14	67	76	81	37	18
2	T	C	G	989	11	0	1	0	0	48	39	0	0	11
3	T	C	A	0	3	0	0	0	0	0	0	26	3	9
4	T	T	G	2	0	0	0	0	0	0	0	0	0	0
5	T	T	A	6	0	0	0	1	0	0	1	0	0	2
6	C	C	G	4	0	0	0	0	0	1	0	0	0	0
7	C	C	A	0	0	0	0	0	0	0	0	1	0	0
8	C	T	G	3	0	0	0	0	0	0	0	0	0	0
No of animals				891	20	20	20	41	7	58	58	54	20	20

a Purebred pigs include Duroc (DU), Landrace (L), Pietrain, Iberian (IB), and wild boar. Numbers after the breed refer to independent lines from the same breed. The Duroc-1 was the line used for the association analysis (Exp 1) and crossbreds in Exp 2 where those used for the validation analysis.

**Table 2:** Desaturation 18∶1/18∶0 ratio and content of 18∶0+18∶1 by batch and *SCD* diplotype in purebred Duroc.

				18∶1/18∶0								18∶0+18∶1 (%)			
				Diplotype				Additive (a) and dominant (d) values				Diplotype			
Batch	Year	n	f(H1)	H1H1	H1H2	H2H2	p-value	a	p-value	d	p-value	H1H1	H1H2	H2H2	p-value
1	2002	109	0.33	3.37^a^	3.34^a^	3.04^b^	<0.001	0.16	0.004	0.14	0.07	53.98	54.55	54.35	0.53
2	2003	71	0.51	3.45^a^	3.19^b^	3.05^b^	0.002	0.20	<0.001	−0.06	0.36	55.03^b^	56.13^a^	55.72^ab^	0.04
3	2003	28	0.46	3.18^a^	2.96^a^	2.65^b^	0.001	0.27	<0.001	0.04	0.62	55.09	55.64	56.14	0.30
4	2006	28	0.57	4.33	3.98	3.70	0.10	0.32	0.038	−0.04	0.88	55.00	54.24	53.90	0.53
5	2006	22	0.55	4.86^a^	4.43^b^	3.75^c^	<0.001	0.55	<0.001	0.12	0.42	55.49	55.84	54.37	0.38
6	2006	109	0.44	6.20^a^	5.83^a^	5.37^b^	<0.001	0.42	<0.001	0.05	0.69	57.10	57.31	56.77	0.55
7	2007	101	0.46	4.92^a^	4.54^b^	4.29^b^	<0.001	0.31	<0.001	−0.07	0.50	56.38	56.94	56.90	0.75
8	2008	66	0.44	5.96^a^	5.12^b^	4.54^b^	<0.001	0.71	<0.001	−0.13	0.56	58.26	56.65	56.64	0.05
9	2008	72	0.38	4.35^a^	3.77^b^	3.50^c^	<0.001	0.43	<0.001	−0.15	0.07	55.46^a^	53.90^b^	54.46^ab^	0.02
10	2010	84	0.39	4.35^a^	4.29^a^	3.89^b^	0.006	0.23	0.011	0.17	0.20	56.91	56.60	56.50	0.80
11	2010	95	0.50	4.34^a^	4.04^b^	3.72^c^	<0.001	0.31	<0.001	0.01	0.87	55.79	55.75	56.30	0.36
12	2011	81	0.46	4.01^a^	3.82^ab^	3.73^b^	0.016	0.14	0.005	−0.05	0.43	57.94	57.67	57.98	0.38
All	–	866	0.44	4.43^a^	4.11^b^	3.78^c^	<0.001	0.33	<0.001	0.00	>0.99	56.10	56.03	55.98	0.81

The haplotype H1 affects the 18∶1/18∶0 ratio in the muscle *gluteus medius* but not the 18∶0+18∶1 content (in percentage of total fatty acids). H1 exerts a consistent favourable additive effect on the desaturation ratio across all time-batches. Analyses were performed both within batch (1 to 12) and across batches (All). Values are expressed as the least square mean for each trait by genotype. Means lacking a common superscript within trait differ (p<0.05). The number of pigs (n) genotyped per batch ranged from 22 to 109. The frequency of the haplotype H1 (f (H1)) by batch ranged from 0.33 to 0.57.

**Figure 5:**
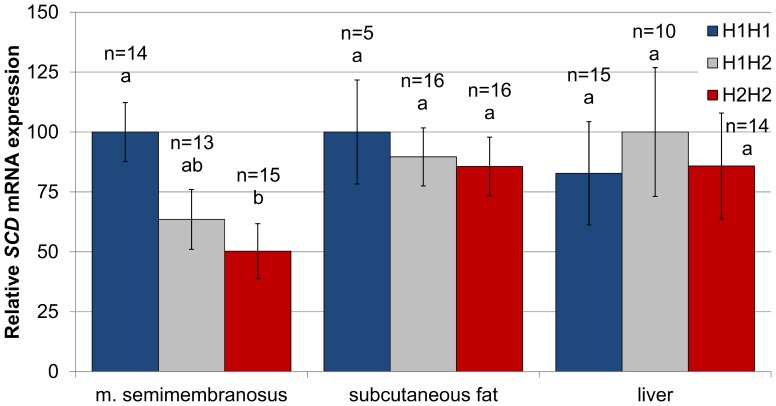
The haplotype H1 upregulates *SCD* mRNA expression in muscle. Pigs H1H1 had higher *SCD* mRNA expression than the H2H2 pigs in muscle *semimembranosus* but not in subcutaneous fat and liver. Values are expressed relative to the mean expression in the diplotype with the greater expression in each tissue. Error bars represent standard errors. Columns lacking a common letter within tissue differ (p<0.05). Haplotype H1 had a favorable additive effect on *SCD* mRNA expression in muscle (24.9±8.2, p<0.01) but not in subcutaneous fat (7.2±12.5, p = 0.57) and liver (−1.5±15.0, p = 0.91).

To assess the functional impact of the haplotype association we analysed the *SCD* mRNA expression in muscle, subcutaneous adipose tissue, and liver across diplotypes. In accordance with the association results, we found that H1H1 animals showed greater *SCD* mRNA expression than H2H2 pigs in muscle (**Figure 5**). Despite the trend was the expected, we were not able to detect significant differences in *SCD* mRNA expression between diplotypes in subcutaneous fat. The haplotype had no effect on the *SCD* mRNA expression in liver.

### Validation and Haplotype Determination

**Figure 6:**
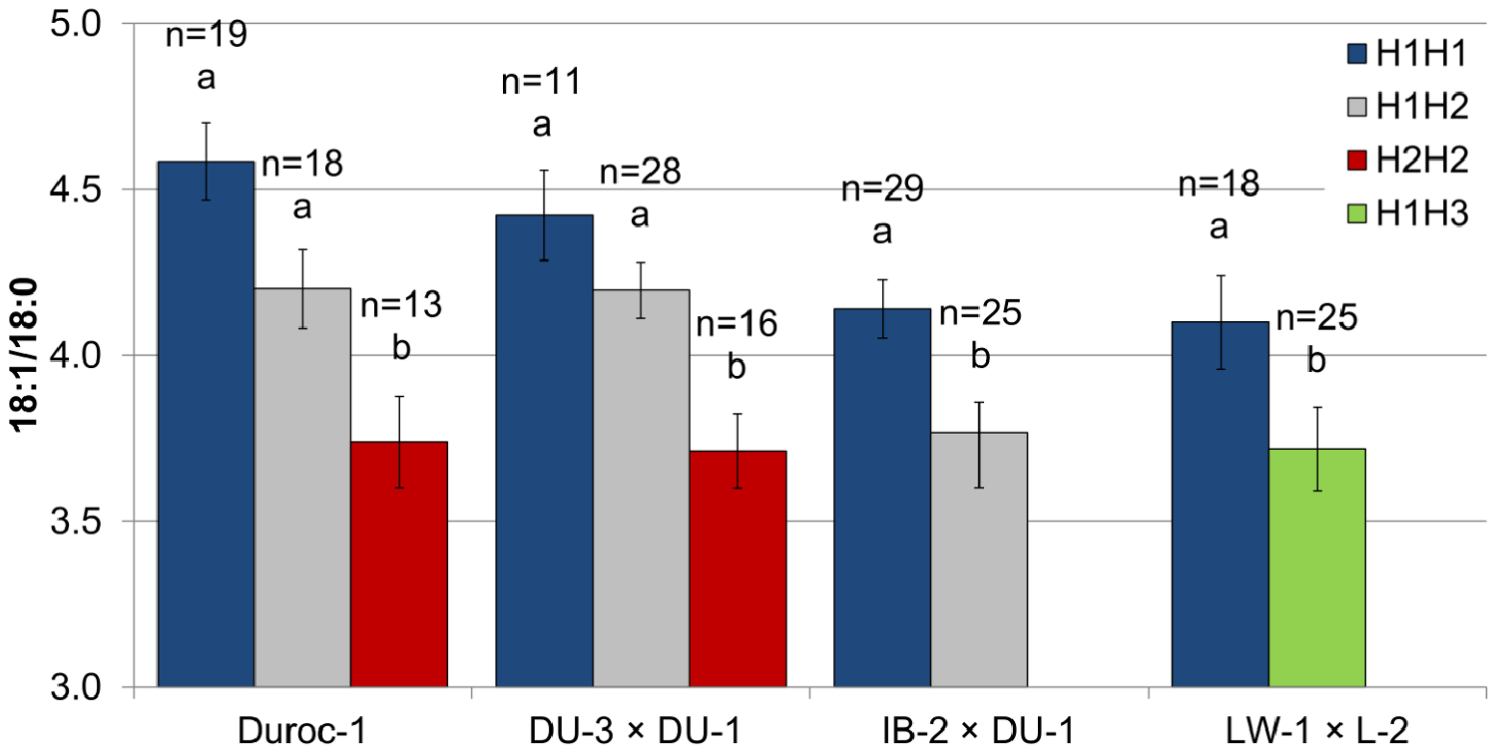
Desaturation ratio by *SCD* diplotype in experimental crossbreds. The effect of *SCD* haplotypes on the 18∶1/18∶0 ratio was validated in three experimental genetic types. Sows from the investigated Duroc line (Duroc-1), which was used as control, were sired by boars from an independent Duroc line (DU-3 × DU-1) and by Iberian boars (IB-2 × DU-1), and their progeny contemporarily compared with Large White × Landrace barrows (LW-1 × L-2). The results confirmed that the H1 haplotype increased the 18∶1/18∶0 ratio in the *gluteus medius* muscle in all genetic types. The H1H1 pigs showed a higher desaturation ratio than H2H2 (0.81 more in Duroc-1 and and 0.61 more in DU-3 × DU-1), H1H2 (0.37 more in IB-2 × DU-1), and H1H3 (0.38 more in LW-1 × L-2) pigs. All LW-1 × L-2 pigs were AA for SNP *g.2281A>G*, thereby excluding this SNP as a causative mutation. Error bars represent standard errors. Columns lacking a common letter within genetic type differ (p<0.05).

We next validated the effect of the haplotypes on experimental Duroc crossbreds (Exp 2; **Table 1**). To that end, Duroc sows from the line used in Exp 1 were mated, in addition to Duroc boars from the same line (genetic type control), to either Duroc boars from a leaner commercial line or to Iberian boars where the H1 haplotype was fixed. Barrows from contemporary offspring of the three mating types were raised in two batches. The Duroc crossbred types reproduced not only the favorable effect of H1 on the 18∶1/18∶0 ratio, but also, when compared to purebred Duroc, replicated the magnitude of the effect as well (**Figure 6**). Thus, the substitution effect of H1 for H2 for 18∶1/18∶0 remained close to 0.40. Moreover, as expected, the H1 variant increased the 16∶1/16∶0 ratio, but did not affect body growth and fatness **(Table S4**).

To refine the haplotype block determination and disentangle which SNP was the responsible of the haplotype effect, we investigated the progeny of two heterozygote C-T-A/T-C-A (H1H3) Large White boars. Barrows from mating these boars with H1H1 Landrace sows were contemporaneously raised with pigs in Exp 2, with the expectation to obtain half of the offspring C-T-A/C-T-A (H1H1) and half C-T-A/T-C-A (H1H3) (i.e., segregating at *g.2108C>T* and *g.2228T>C*, while fixed at *g.2281A>G*). The haplotype segregation was as expected (**Table 1**). This experiment showed that, similarly to contemporaneously purebred and crossbreds Duroc in Exp 2, H1H1 Large White × Landrace barrows still displayed a greater 18∶1/18∶0 ratio than heterozygotes carrying the H3 haplotype (**Figure 6**). As with other genetic types in Exp 2, the haplotype in Large White × Landrace had no effect on traits other than those directly affected by SCD (**Table S4**). Importantly, the results of this last validation experiment allow us to exclude SNP *g.2281A>G* as the causative mutation of the effect over the desaturation index.

### *In silico* Analysis of *SCD* Promoter Polymorphisms

To assess if polymorphisms in the promoter region could affect *SCD* expression through the disruption of transcription factor binding sites, a computer-assisted identification of potential *cis*-acting DNA-sequence motifs was carried out. As a first step, we analyzed in parallel the promoter region (−500 to +100 from the transcriptional start site) of human, cow, pig, and sheep *SCD* gene with the view of identifying common regulatory modules. The promoter sequence displays stretches of strong conservation between these four species interspersed with fragments of lower conservation (**Figure S1**). A conserved polyunsaturated fatty acid response element (PUFA-RE), which includes a sterol regulatory element (SRE), two CCAAT boxes (NF-Y) and two nuclear factor (NF)-1 binding sites, has been described approximately at positions −450/−550, which is highly conserved between species [26]–[29] (**Figure S1**) and is essential for correct *SCD* gene regulation by PUFA and cholesterol [26], [27]. The transcription factor SRE binding protein-1 down-regulates *SCD* expression through the interaction with the SRE element in this regulatory region [26]. In addition to the PUFA-RE element, our *in silico* analyses revealed a conserved peroxisome proliferator-activated receptor gamma (PPARG) motif at position −400/−420. Another region containing many potential binding motifs lays in the sequence around the *g.2228T>C* polymorphism, about 40 bp upstream of the TATA-box. Several transcription factor-binding motifs partially overlap in this region. There is a conserved PPARG and NF-1 motif on the negative strand, which lay adjacent to a CCAAT/enhancer binding protein motif (C/EBP) in cow, sheep and humans. However, our analysis failed to recognize this C/EBP motif in the pig sequence, although it has been postulated before [27]. In pig this motif is replaced by two PPARG binding sites, a half-site in the positive strand and a full homodimer motif with 3-bp inter half-site spacing between inverted repeats (IR3) where PPARG binds to both complementary strands (**Table S5**). In addition, bridging these PPARG sites together, there are two binding motifs for the retinoid X receptor and the retinoic acid receptor α (RXR:RARα) response elements (direct repeats with 1-bp (DR1) and 5-bp (DR5) spacer sequence, respectively). The *g.2228T>C* polymorphism lies in the core of the IR3 motif in the positive strand and at the end of the DR5 element (**Figure S1**).

## Discussion

We have shown that the C-T haplotype at SNPs *g.2108C>T* and *g.2228T>C* in the promoter region of the *SCD* gene increases fatty acid desaturation in muscle and subcutaneous fat, in line with some previous findings in Duroc [21]. The third polymorphism screened in the promoter (*g.2281A>G*) was excluded as a causal by the results in our external validation experiment (Exp 2), where Large White × Landrace pigs were all homozygous for this SNP but still presented the same effect over the desaturation index. Conversely, as in all the screened populations SNPs *g.2108C>T* and *g.2228T>C* were in almost complete linkage disequilibrium, we were not able to disentangle which one of the two is the causative mutation. However, considering the results from a Landrace × Korean native pig intercross in which these two SNPs segregated independently [22], we can conclude that fatty acid composition was associated to an haplotype comprised, in its 5′ extreme, not beyond the g.2109 position and, in its 3′ end, not past the g.2280 nucleotide (**Figure 2**). However, no other mutations have been described in this short 173 bp region in other studies which have extensively sequenced the *SCD* promoter in independent Duroc lines [21]–[24], including the present study. In contrast, the *g.2228T>C* SNP is common to all the studies which have found a significant relationship between the *SCD* promoter genotype and fatty acid composition [21], [22]. Taken together, these findings strongly support that allele T at *g.2228T>C* is the causative mutation leading to increased fatty acid desaturation. Interestingly, this allele is virtually absent in the Asian breeds [30] and, in contrast, almost fixed in other breeds, including Landrace, Pietrain, Iberian, and wild boar (**Table 1**; [24]). This explains why whole-genome analyses based on these latter breeds failed to identify *SCD* as a positional candidate gene for fatty acid composition. It remains unclear why Duroc is the only breed where *g.2228T>C* is segregating at intermediate frequencies.

In pig, the *g.2228T>C* SNP is positioned at 58 nt from the *SCD* transcription start site, in a stretch of moderate sequence conservation with cow, sheep, and human *SCD* sequences (**Figure S1**). *In silico* analysis of this region has identified several overlapping putative transcription factor binding sites, some of which are unique to the pig promoter and contain the T>C mutation at position g.2228 (**Figure 2**). Among them, there are the two putative DR1 and DR5 retinoic acid response elements overlapping to two PPARG motifs. The DR1 is a high affinity response element for RXR:RARα and PPARG:RARα heterodimers [31], which regulate gene expression in response to their ligands, all-trans or 9-cis retinoic acid. A recent genome-side study revealed that the consensus PPARG/RXRα DR1-binding motifs co-localized at nearly all locations tested [31]. Bioinformatics analysis also revealed C/EBP-binding motifs in the vicinity of most PPARG-binding sites in genes induced in adipogenesis. Thus, PPARG and C/EBP factors cooperatively regulate adipocyte-specific gene expression by adjacent binding [31]. Unlike other authors [27], [29], we failed to identify the C/EBP motif in the pig promoter, although it has been described for instance in the human, mouse, sheep and cow promoters [26], [28], [32].

By which mechanism the *g.2228T>C* polymorphism enhances *SCD* expression is unknown, although we can postulate three possible scenarios. In the first one, the T>C mutation, which affects a core nucleotide of the PPARG homodimer motif, might alter the PPARG binding affinity to this site. In a second scenario, the mutation might alter the affinity of the RXR:RARα to their target DNA motifs, enhancing or repressing transcription depending on the nature of the motif. And lastly, our third possible scenario relies on the cooperative binding between RXR:RARα and PPARG sites, which is a wide-spread feature in the genome [31], and that the g.2228T>C mutation alters the relative affinity of one or both of these regulatory partners. This mechanism is additionally fine-tuned by the availability and concentration of different ligands, which not only modulates their affinity for the DNA binding sites, but also their ability to interact with other co-activators, thus defining their enhancing or inhibitory action over gene expression [33].

In this regard, we were able to prove increased *SCD* transcription in TT pigs as compared to CC pigs in muscle, indicating that higher product-to-precursor ratios in pigs carrying the allele T are a consequence of increased *SCD* expression rather than a more active version of the protein, as the two main haplotypes did not differ in the coding region sequence. Moreover, our results indicate that the enhanced activity of the allele T of the *SCD* gene is tissue-specific, with preference for muscle, and substrate-specific, with preference for 18∶0 rather than 16∶0. In contrast to subcutaneous fat, IMF is less sensitive to dietary fat and, conversely, more prone to endogenous fatty acid synthesis and remodeling, particularly regarding 18∶1 [8]. Therefore, differences across *SCD* genotypes are expected to be better accounted for in muscle than in the subcutaneous tissue. We have seen in a previous experiment that genetic selection of pigs against fatness led to differential responses in SCD protein expression in muscle and subcutaneous adipose tissue [34]. The tissue-specific behavior of the pig *SCD* gene is also shown by distinct patterns of CpG methylation in the proximal promoter in muscle as compared to subcutaneous fat [35]. In contrast, the *SCD* promoter genotypes had no impact on liver fatty acid composition, which is in line with the fact that, in pigs, the adipose tissue, and not the liver, is the principal site of de novo fatty acid synthesis [36]. Moreover, in liver, genes encoding for fatty acid remodeling enzymes, such as *SCD*, respond differently to steroid hormone stimulation that genes involved in the fatty acid biosynthesis. For instance, unlike fatty acid synthase or malic enzyme gene, the hepatic pig *SCD* gene undergoes a negative response to thyroid hormone occurring through a thyroid receptor response element located downstream the g.2228T>C [37]. Although indirectly, the results here also indicate that the expected extra SCD produced by allele T prefers 18∶0 rather than 16∶0 as a substrate. Thus, we observed that allele T has a consistent negative side effect on the 18∶0/16∶0 ratio. Because there is no reason for differential dietary deposition of fatty acids across genotypes (they were subjected to the same diet), a likely interpretation is that 18∶0 is consumed more steadily than 16∶0, which may occur if SCD desaturates 18∶0 to 18∶1 more efficiently than 16∶0 to 16∶1 [9]. Comparison of the means of 16∶0, 16∶1, 18∶0, and 18∶1 for the two extreme genotypes (Table S2) shows that, in *gluteus medius*, TT homozygotes desaturate 10.9% more 18∶0 than the CC but only 2.1% more C16∶0. As for the subcutaneous fat, these values were 8.5% and 3.0%, respectively, thereby reproducing the same pattern. The substrate specificity may be due to different SCD isoforms [12]. A recent update of the pig *SCD* annotation in *Ensembl*, corresponding to assembly Sscrofa10.2 release 72 (performed on June 2013) reported three new isoforms for the *SCD* gene, bringing the total number to four. They are translated into four different peptides. The tissue-dependent expression of these isoforms is another level of complexity of the activity of the *SCD* expression that has not yet been explored in pigs.

In addition, the regulation of *SCD* expression is a complex phenomenon. The intracellular concentration of desaturases fluctuates in response to a large number of effectors including hormonal and dietary factors [11]. However, the influence of dietary treatment on muscle fatty acid composition is not evident [38], likely because deposition of dietary fat can be offset by endogenous synthesis. It has been shown experimentally in pigs that a reduced protein diet enhances *SCD* expression in muscle but not in subcutaneous adipose tissue [39]. The favorable effect of the allele T on 18∶1/18∶0, although consistent, varied across batches. A key component of all the environmental factors accounted for in the batch effect is the diet. We have seen that there is a negative relationship of the additive effect of this allele in muscle with dietary protein (R^2^ = 0.38, p<0.05). In contrast, the dietary 18∶1/18∶0 ratio exerted a positive effect on the additive effect of allele T in muscle (R^2^ = 0.39, p<0.05). These effects were not detected in the subcutaneous fat. Overall, these findings not only give additional evidence that the effect of the *SCD* genotypes is most noticeable in muscle, but also that it is tuned by the diet. In this regard, an interesting topic for future research will be to study the effect of these haplotype variants in pigs subjected at diets differing in vitamin A, or some other metabolic precursor of retinoic acid. In line with two of our hypothetical scenarios, it has been shown experimentally that retinoic acid inhibits porcine preadipocyte differentiation by upregulating RAR and downregulating RXR [40] but the effects of dietary vitamin A on IMF content and fatty acid composition in pigs are scarce and inconclusive [41], with results depending on the genetic type [42]. The study of the g.2228T>C mutation may contribute to unravel the biological causes of the interaction between dietary vitamin A and gene expression. Moreover, because the RAR and RXR mRNA levels decline with age [43], it may also help to explain the favorable evolution of the 18∶1/18∶0 ratio with age [8].

We provide evidence that there exists genetic variation in the *SCD* gene with the potential to increase MUFA content in pork. Strict values on fatty acid content are becoming a common feature in regulations for foods bearing nutritional or health claims concerning fat properties. The MUFA content can be also subjected to such regulations. Selective lipid deposition in meat animals is a relevant issue not only in terms of animal agriculture but also in biomedicine. Evidence is also emerging indicating the existence of allelic variations in the human *SCD* gene affecting enzyme activity and, consequently, disease risk factors [10]. Therefore, research in meat animals may well not only lead to a new understanding of the regulation of lipid metabolism [36] but also to integrate agriculture science, nutrition, and pharmacology for improved treatment of important chronic diseases [44].

## Materials and Methods

### Ethics Statement

The experimental protocol was approved by the Committee on the Ethics of Animal Experiments of the University of Lleida.

### Animals and Tissue Sampling

The association analysis (Exp 1) was done using genomic DNA and phenotypic data of twelve batches (n = 891) of purebred Duroc barrows from the line described in [25] (Duroc-1; **Table 1**). In two of these batches, crossbred Duroc (DU-3 × DU-1), Duroc × Iberian (IB-2 × DU-1), and Large White × Landrace (LW-1 × L-2) barrows (Exp 2) were contemporaneously raised to Duroc-1 barrows, for validation purposes (n = 170; **Table 1**). Pigs in the same batch were raised from 75 days of age until slaughter at 205 days in the same farm under identical conditions. All batches were managed following the same standard protocol for data recording and tissue sampling [23]. Barrows had *ad libitum* access to commercial diets. From 160 days of age onwards they were fed a pelleted finishing diet with an average composition of 16.9% crude protein, 6.6% fiber, and 6.7% fat (16∶0: 20.8%; 18∶0: 7.1%; 18∶1: 35.4%; 18∶2: 27.4%). In two of the Duroc batches at 180 days of age three 10-mL samples of blood per barrow were obtained between 8 and 10 a.m. after an overnight fast. All pigs were slaughtered in the same commercial abattoir, where lean content and other carcass traits were measured by using an on-line ultrasound automatic scanner. Immediately after slaughter, samples of the *semimembranosus* muscle, subcutaneous adipose tissue at the level of the third and fourth ribs, and liver were collected, snap-frozen, and stored at −80°C. After chilling for about 24 h at 2°C, a sample of the *gluteus medius* muscle was excised from the left side ham, vacuum packaged, and stored at −80°C. Finally, we used genomic DNA representing European wild boar and several domestic breeds of pigs and commercial crossbreds for monitoring haplotype segregation.

### Fatty Acid and Blood Lipid Indicator Analysis

A representative aliquot from the pulverized freeze-dried tissue was used for fat analysis. Fat content and fatty composition was determined in duplicate by quantitative determination of the individual fatty acids by gas chromatography [45]. Fatty acid methyl esters were directly obtained by transesterification using a solution of 20% boron trifluoride in methanol and then determined by gas chromatography using a capillary column SP2330 (30 m × 0.25 mm, Supelco, Bellefonte, PA). Quantification was carried out through area normalization after adding into each sample 1,2,3-tripentadecanoylglycerol as internal standard. Fatty acids were identified by comparing their relative retention times with those of the external standard and confirmed by comparing their mass spectra to the computer library of the GC/MS database Wiley 275.L and NBS 75 K.L. The proportion of individual fatty acids, as well as that of SFA (14∶0; 16∶0; 18∶0; and 20∶0), MUFA (16∶1; 18∶1; and 20∶1), and PUFA (18∶2; 18∶3; 20∶2; and 20∶4), were calculated as percentages relative to total fatty acid content. Blood triglycerides, cholesterol, leptin and insulin-like growth factor-1 were determined using available kits [46].

### Nucleic Acids Isolation

Genomic DNA was isolated from freeze-dried muscle samples using standard protocols [47]. Total RNA was isolated from fat, liver and *semimembranosus* muscle. Samples (50 mg) were homogenized in 1 mL of TRI Reagent (Sigma-Aldrich, Madrid, Spain) using a mechanical rotor (IKA Werke, Staufen, Germany) following the manufacturer’s instructions.

### Sequencing of Promoter and Exonic Regions of the Pig *SCD* Gene

Based on genomic and cDNA sequences (GenBank accession numbers AY487830 and NM_213781, respectively) primers were designed in order to amplify and sequence 780 bp of the *SCD* proximal promoter and the entire exonic regions of the gene. Seven primer sets were designed with the Primer3Plus online oligonucleotide design tool (http://primer3plus.com) [48] (**Table S6**). The promoter and 3′ non-coding region were amplified from approximately 60 ng of genomic DNA from twelve Duroc pigs selected to represent extreme levels of oleic acid in *gluteus medius*. PCR reaction of a final volume of 25 µL contained 200 nM of each primer, 160 mM dNTPs, 3 mM MgCl_2_, and 0.4 U of *Taq* DNA polymerase (Biotools, Madrid, Spain). PCR conditions were as follows: 95°C for 5 minutes, 35 cycles of 95°C for 20 sec, annealing temperature as in **Table S6** for 40 sec, and 72°C for 90 sec, and completed by an extension step at 72°C for 5 min. The 5′ non-coding and coding regions were amplified using the same reaction and cycling conditions from total RNA of *semimembranosus* muscle retrotranscribed to cDNA as indicated in the *Gene Expression Analysis* section. PCR amplicons were sequenced on an ABI-3100 capillary sequencer (Applied Biosystems, Foster City, CA) with the BigDye Terminator v3.1 Cycle Sequencing Kit (Applied Biosystems). Sequences were aligned with the ClustalW alignment tool (http://www.ebi.ac.uk/Tools/msa/clustalw2/) and compared to identify polymorphic sites. All sequences have been submitted to the GenBank data base (accession numbers KC736975 and KC736976).

### Genotyping the Pig *SCD* Promoter

Three *SCD* promoter polymorphisms (*AY487830:g.2108C>T*, *g.2228T>C* and *g.2281A>G*) were genotyped with allele discrimination assays (Custom TaqMan SNP Genotyping Assays, Applied Biosystems) using the primers and probes described in **Table S7**. For all of them, 15 ng of genomic DNA were used in 8 µL reactions containing 1x TaqMan Universal PCR Master Mix (Applied Biosystems) and 900 nM primers and 200 nM probes. Cycling conditions were as follows: Initial denaturation at 95°C for 10 min and 40 cycles at 93°C for 5 sec and 60°C for 1 min.

### Gene Expression Analysis

*SCD* expression levels were measured by quantitative real-time PCR (qPCR) in *semimembranosus* muscle, subcutaneous fat, and liver and from a subset of 45 animals representing diplotypes H1H1, H1H2, and H2H2. Total RNA (1 µg) was treated with Turbo DNA-free DNase (Ambion, Austin, TX) according to the manufacturer’s protocol and retrotranscribed with 0.5 pmol of random hexamers using 100 U of MuMLV reverse transcriptase (Fermentas, St. Leon-Rot, Germany) at 25°C for 10 min, 42°C for 1 h and 70°C for 10 min. cDNA was diluted 1∶10 in DEPC-treated H_2_O prior to qPCR analysis. Primers, PCR conditions and data normalization was conducted as in [49].

### Estimating Haplotype Effects

The haplotype effect was estimated within tissue using a linear model including the diplotype and the batch (JMP 8, SAS Institute Inc., Cary, NC). The age at slaughter and fat content were tested as covariates in the model. The haplotype additive (a) and dominant (d) effects were tested replacing the diplotype effect by the covariates a (1; 0; −1) and d (0; 1; 0) for diplotypes H1H1, H1H2, and H2H2, respectively. The effects of the diplotype and covariates were tested using the F-statistic and the differences among diplotypes were contrasted with the Tukey-HSD test. The batch was removed from the model when results were expressed on a batch basis (Exp 1). The haplotype effect in the validation experiment (Exp 2) was estimated within genetic type using the same procedure. In IB-2 × DU-1 and LW-1 × L-2 crossbreds, the sire effect was included in the model because only two IB-2 and LW-1 sires were used. A paired t-test was used for comparing homozygote siblings. The additive fraction of the genetic variance accounted for by the diplotype was calculated as 2pqa^2^ [50] divided by the additive genetic variance. The genetic variance for fatty acids and their ratios were estimated using the approach in [25] and univariate animal models including the full pedigree since 1991.

### *In silico* Analysis of the *SCD* Promoter

To characterize the *SCD* promoter, a computer-assisted identification of putative promoter/enhancer elements was performed using the GENOMATIX software suite (Genomatix Software GmbH) [51]. Genomatix Matrix Library 8.3 was used with a core similarity threshold of 0.85 and an optimized matrix similarity threshold (program default). The Gene2Promoter application was used to retrieve the *SCD* promoter from pig, human, cow, and sheep. Common transcription factor binding motifs were explored using the CommonTF, DiAlignTF and MatInspector applications for pattern search and analysis.

## Supporting Information

Figure S1 **Comparative promoter sequence between cow, pig, sheep and human** ***SCD*** **gene.** **Panel (A)** depicts a sequence alignment of a 700 bp homologous 5′ flanking sequence of the gene using ClustalW (http://www.ebi.ac.uk/Tools/msa/clustalw2/). The conserved PUFA response element including a sterol response element (SRE), two CCAAT-box (NF-Y), two nuclear factor (NF)-1 and one stimulator protein 1 (SP1) binding site is boxed. Other common motifs (TATA-box, NF-1 and PPARG) are also indicated along with the position of the three pig promoter SNPs genotyped. Several putative transcription factor binding sites close to the *g.2228T>C* polymorphism are depicted in the four species; these include a putative CCAAT enhancer binding protein (C/EBP) element, NF-1, two PPARG binding sites, and two RAR:RXR motifs (DR1 and DR3). The diagram in **Panel (B)** represents the potential binding of these transcription factors in the sequence around the *g.2228T>C* polymorphism.
(TIF)

Table S1 **Description of the polymorphisms identified at** ***SCD*** **gene.** Eighteen polymorphisms in the *SCD* gene were found to be segregating in the investigated Duroc population by comparing the DNA sequence of six pigs with extreme high and low values for oleic acid content in *gluteus medius* muscle. Position numbering is relative to the translation start codon and the genomic sequence AY487830. Three of the polymorphisms are single-nucleotide substitutions in the promoter region.
(DOCX)

Table S2 **Carcass weight, fat content, and fatty acid composition by** ***SCD*** **diplotype and fat tissue in purebred Duroc.** The haplotype H1 showed a favorable effect on fatty acid compositional traits resulting from increased SCD activity (16∶1/16∶0, 18∶1/18∶0, MUFA/SFA, 18∶1, 16∶1, and MUFA) and no effect on fat content-related traits (carcass weight, lean content, intramuscular fat content, 16∶0+16∶1, 18∶0+18∶1, and SFA+MUFA). This pattern was more evident in muscle than in subcutaneous fat. Values are expressed as the least square mean (± standard error) for each trait by diplotype. Means lacking a common superscript within trait differ (p<0.05).
(DOCX)

Table S3 **Blood lipid indicators by** ***SCD*** **diplotype in purebred Duroc.** The diplotype did not affect (p<0.05) blood plasma lipid indicators at 180 d. Values are expressed as the least square mean (± standard error) for each trait by diplotype.
(DOCX)

Table S4 **Carcass weight, fat content, and fatty acid composition by** ***SCD*** **diplotype in experimental crossbred pigs.** The haplotype H1 showed a favorable effect on 16∶1/16∶0 and 18∶1/18∶0 ratios and no effect on fat content-related traits (carcass weight, lean content, intramuscular fat content, 16∶0+16∶1, and 18∶0+18∶1). Values are expressed as the least square mean (± standard error) for each trait by diplotype. Means lacking a common superscript within trait differ (p<0.05).
(DOCX)

Table S5 **Positioning of the putative transcription factor binding sites in the proximal promoter of the pig** ***SCD*** **gene.** Results from the in silica analysis performed with the MatInspector Genomatix program. The putative PPARG, RAR:RXR and NF-1 motifs around the *AY487830:g.2228T>C* SNP are highlighted.
(XLSX)

Table S6 **Sequence of DNA primers used in the characterisation of the porcine** ***SCD*** **gene.** A list of the primers used to amplify and sequence seven fragments of the porcine *SCD* gene encompassing 780 bp of the promoter promoter and the entire coding and 5′ and 3′ non-coding regions (3UTR). The annealing temperature used in the PCR cycling program is also indicated.
(DOCX)

Table S7 **Primers used for genotyping the three single nucleotide polymorphisms (SNPs) in the porcine** ***SCD*** **gene promoter with an allelic discrimination assay.**
(DOCX)
