# Supplementary figures and images for: A Functional Variant in the Stearoyl-CoA Desaturase Gene Promoter Enhances Fatty Acid Desaturation in Pork
Source: PLoS One. 2014 Jan 20;9(1):e86177. doi: 10.1371/journal.pone.0086177 (PMC3896438; doi:10.1371/journal.pone.0086177)

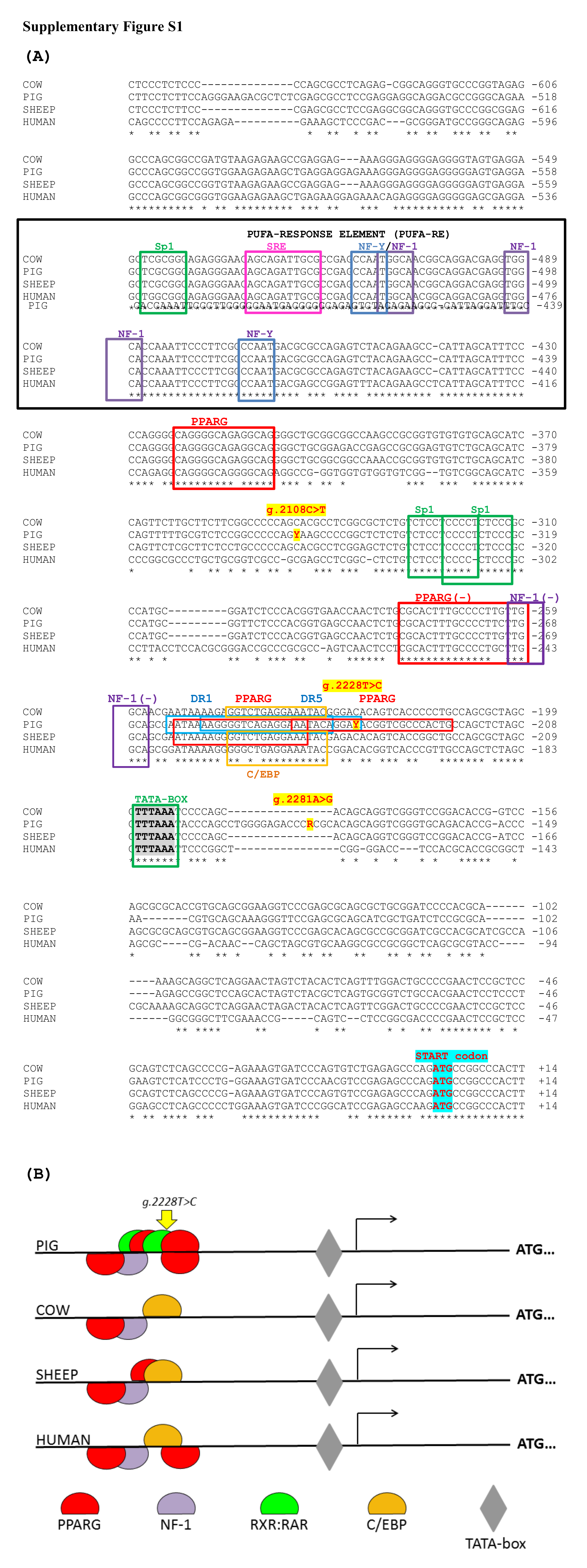

Supplement: Figure S1 — Comparative promoter sequence between cow, pig, sheep and human SCD gene. Panel (A) depicts a sequence alignment of a 700 bp homologous 5′ flanking sequence of the gene using ClustalW (http://www.ebi.ac.uk/Tools/msa/clustalw2/). The conserved PUFA response element including a sterol response element (SRE), two CCAAT-box (NF-Y), two nuclear factor (NF)-1 and one stimulator protein 1 (SP1) binding site is boxed. Other common motifs (TATA-box, NF-1 and PPARG) are also indicated along with the position of the three pig promoter SNPs genotyped. Several putative transcription factor binding sites close to the g.2228T>C polymorphism are depicted in the four species; these include a putative CCAAT enhancer binding protein (C/EBP) element, NF-1, two PPARG binding sites, and two RAR:RXR motifs (DR1 and DR3). The diagram in Panel (B) represents the potential binding of these transcription factors in the sequence around the g.2228T>C polymorphism. (TIF) [file pone.0086177.s001.tif]
