# Supplementary material for: A Functional Variant in the Stearoyl-CoA Desaturase Gene Promoter Enhances Fatty Acid Desaturation in Pork
Source: PLoS One. 2014 Jan 20;9(1):e86177. doi: 10.1371/journal.pone.0086177 (PMC3896438; doi:10.1371/journal.pone.0086177)
Supplement: Table S1 — Description of the polymorphisms identified at SCD gene. Eighteen polymorphisms in the SCD gene were found to be segregating in the investigated Duroc population by comparing the DNA sequence of six pigs with extreme high and low values for oleic acid content in gluteus medius muscle. Position numbering is relative to the translation start codon and the genomic sequence AY487830. Three of the polymorphisms are single-nucleotide substitutions in the promoter region. (DOCX) [file pone.0086177.s002.docx]

**Table S1. Description of the polymorphisms identified at *SCD* gene**. Eighteen polymorphisms in the *SCD* gene were found to be segregating in the investigated Duroc population by comparing the DNA sequence of six pigs with extreme high and low values for oleic acid content in *gluteus medius* muscle. Position numbering is relative to the translation start codon and the genomic sequence AY487830. Three of the polymorphisms are single-nucleotide substitutions in the promoter region.

| **Polymorphisms in AY487830** | **Gene Region** | **Sequence**  **change** | **Position + ATG** | **H1** | **H2** |
| --- | --- | --- | --- | --- | --- |
| *g.2108C>T* | Promoter | Y | -353 | C | T |
| *g.2228T>C* | Promoter | Y | -233 | T | C |
| *g.2281A>G* | Promoter | R | -180 | A | G |
| *g.14924G>A* | 3’UTR | R | +1382 | G | A |
| *g.14981C>T* | 3’UTR | Y | +1439 | C | T |
| *g.15013T>C* | 3’UTR | Y | +1471 | T | C |
| *g.15060A>G* | 3’UTR | R | +1518 | A | G |
| *g.15109A>G* | 3’UTR | R | +1566 | A | G |
| *g.15115_15119insATGG* | 3’UTR | ins (ATGG) | +1572 | - | ATGG |
| *g.15157C>T* | 3’UTR | Y | +1618 | C | T |
| *g.15294G>A* | 3’UTR | R | +1755 | G | A |
| *g.16195G>A* | 3’UTR | R | +2656 | G | A |
| *g.16617A>G* | 3’UTR | R | +3078 | A | G |
| *g.16623A>G* | 3’UTR | R | +3084 | A | G |
| *g.16663T>C* | 3’UTR | Y | +3124 | T | C |
| *g.17305G>C* | 3’UTR | S | +3766 | G | C |
| *g.17313G>T* | 3’UTR | K | +3774 | G | T |
| *g.17437A>C* | 3’UTR | M | +3898 | A | C |
