# Supplementary material for: A Functional Variant in the Stearoyl-CoA Desaturase Gene Promoter Enhances Fatty Acid Desaturation in Pork
Source: PLoS One. 2014 Jan 20;9(1):e86177. doi: 10.1371/journal.pone.0086177 (PMC3896438; doi:10.1371/journal.pone.0086177)
Supplement: Table S2 — Carcass weight, fat content, and fatty acid composition by SCD diplotype and fat tissue in purebred Duroc. The haplotype H1 showed a favorable effect on fatty acid compositional traits resulting from increased SCD activity (16∶1/16∶0, 18∶1/18∶0, MUFA/SFA, 18∶1, 16∶1, and MUFA) and no effect on fat content-related traits (carcass weight, lean content, intramuscular fat content, 16∶0+16∶1, 18∶0+18∶1, and SFA+MUFA). This pattern was more evident in muscle than in subcutaneous fat. Values are expressed as the least square mean (± standard error) for each trait by diplotype. Means lacking a common superscript within trait differ (p<0.05). (DOCX) [file pone.0086177.s003.docx]

**Table S2. Carcass weight, fat content, and fatty acid composition by *SCD* diplotype and fat tissue in purebred Duroc.** The haplotype H1 showed a favorable effect on fatty acid compositional traits resulting from increased *SCD* activity (16:1/16:0, 18:1/18:0, MUFA/SFA, 18:1, 16:1, and MUFA) and no effect on fat content-related traits (carcass weight, lean content, intramuscular fat content, 16:0+16:1, 18:0+18:1, and SFA+MUFA). This pattern was more evident in muscle than in subcutaneous fat. Values are expressed as the least square mean (± standard error) for each trait by diplotype. Means lacking a common superscript within trait differ (p<0.05).

|  | |  | **Diplotype** | | | |
| --- | --- | --- | --- | --- | --- | --- |
| **Trait** | |  | **H1H1** | **H1H2** | **H2H2** | **p-value** |
| No of pigs | |  | 166 | 435 | 268 | - |
| Age at sampling (days) | |  | 212.1 | 212.0 | 211.9 | - |
| Carcass weight (kg) | |  | 95.7±0.8 | 96.9±0.5 | 96.7±0.6 | 0.38 |
| Backfat depth (mm) | |  | 22.6±0.3 | 23.1±0.2 | 22.9±0.2 | 0.33 |
| Lean content (%) | |  | 43.9±0.4 | 43.3±0.2 | 43.5±0.3 | 0.30 |
| M. *gluteus medius* | |  |  |  |  |  |
|  | No of pigs |  | 167 | 432 | 267 | - |
|  | IMF (% dry matter) |  | 16.57±0.37 | 16.42±0.23 | 16.55±0.30 | 0.91 |
|  | 16:1 (%) |  | 4.10±0.05^a^ | 3.76±0.03^b^ | 3.50±0.04^c^ | <0.001 |
|  | 16:1/16:0 (×100) |  | 18.04±2.46^a^ | 16.18±1.58^b^ | 14.90±2.00^c^ | <0.001 |
|  | 16:0 + 16:1 (%) |  | 27.32±0.11 | 27.24±0.07 | 27.16±0.09 | 0.52 |
|  | 18:1 (%) |  | 45.38±0.16^a^ | 44.71±0.10^b^ | 43.97±0.13^c^ | <0.001 |
|  | 18:1/18:0 |  | 4.43±0.04^a^ | 4.11±0.03^b^ | 3.78±0.03^c^ | <0.001 |
|  | 18:0 + 18:1 (%) |  | 56.10±0.15 | 56.03±0.10 | 55.98±0.12 | 0.81 |
|  | MUFA (%) |  | 50.30±0.17^a^ | 49.27±0.11^b^ | 48.27±0.14^c^ | <0.001 |
|  | MUFA/SFA |  | 1.44±0.01^a^ | 1.37±0.01^b^ | 1.31±0.01^c^ | <0.001 |
|  | SFA + MUFA (%) |  | 86.02±0.17 | 85.88±0.11 | 85.75±0.14 | 0.44 |
| M. *longissimus dorsi* | |  |  |  |  |  |
|  | No of pigs |  | 50 | 165 | 98 | - |
|  | IMF (% dry matter) |  | 12.88±0.47 | 12.96±0.26 | 12.47±0.34 | 0.51 |
|  | MUFA (%) |  | 51.43±0.26^a^ | 50.23±0.14^b^ | 49.22±0.19^c^ | <0.001 |
|  | MUFA/SFA |  | 1.40±0.01^a^ | 1.32±0.01^b^ | 1.27±0.01^c^ | <0.001 |
|  | SFA + MUFA (%) |  | 88.76±0.31 | 88.73±0.17 | 88.49±0.23 | 0.67 |
| M. *semimembranosus* | |  |  |  |  |  |
|  | No of pigs |  | 43 | 86 | 67 | - |
|  | IMF (% dry matter) |  | 10.22±0.70 | 10.01±0.54 | 11.03±0.58 | 0.36 |
|  | MUFA (%) |  | 49.78±0.70 | 48.32±0.53 | 47.94±0.57 | 0.11 |
|  | MUFA/SFA |  | 1.48±0.05 | 1.44±0.04 | 1.39±0.04 | 0.37 |
|  | SFA+ MUFA (%) |  | 83.73±0.62 | 82.88±0.47 | 83.34±0.51 | 0.51 |
| Subcutaneous fat | |  |  |  |  |  |
|  | No of pigs |  | 56 | 124 | 80 | - |
|  | 16:1 (%) |  | 2.20±0.04 | 2.18±0.03 | 2.08±0.03 | 0.029 |
|  | 16:1/16:0 (×100) |  | 10.54±2.79 | 10.32±1.96 | 9.78±2.38 | 0.07 |
|  | 16:0 + 16:1 (%) |  | 23.86±0.22 | 24.10±0.15 | 24.17±0.19 | 0.53 |
|  | 18:1 (%) |  | 44.87±0.28^a^ | 43.76±0.19^b^ | 43.41±0.24^b^ | <0.001 |
|  | 18:1/18:0 |  | 4.38±0.11^a^ | 4.16±0.07^ab^ | 3.90±0.09^b^ | 0.002 |
|  | 18:0 + 18:1 (%) |  | 55.74±0.22^a^ | 54.95±0.16^b^ | 55.16±0.19^ab^ | 0.018 |
|  | MUFA (%) |  | 48.23±0.29^a^ | 47.04±0.21^b^ | 46.56±0.25^b^ | <0.001 |
|  | MUFA/SFA |  | 1.47±0.03^a^ | 1.41±0.02^ab^ | 1.36±0.03^b^ | 0.031 |
|  | SFA + MUFA (%) |  | 82.31±0.26 | 81.78±0.18 | 82.02±0.22 | 0.24 |
