# Supplementary material for: A Functional Variant in the Stearoyl-CoA Desaturase Gene Promoter Enhances Fatty Acid Desaturation in Pork
Source: PLoS One. 2014 Jan 20;9(1):e86177. doi: 10.1371/journal.pone.0086177 (PMC3896438; doi:10.1371/journal.pone.0086177)
Supplement: Table S3 — Blood lipid indicators by SCD diplotype in purebred Duroc. The diplotype did not affect (p<0.05) blood plasma lipid indicators at 180 d. Values are expressed as the least square mean (± standard error) for each trait by diplotype. (DOCX) [file pone.0086177.s004.docx]

**Table S3. Blood lipid indicators by *SCD* diplotype in purebred Duroc.** The diplotype did not affect (p<0.05) blood plasma lipid indicators at 180 d. Values are expressed as the least square mean (± standard error) for each trait by diplotype.

|  | |  | **Diplotype** | | | |
| --- | --- | --- | --- | --- | --- | --- |
| **Trait^1^** | |  | **H1H1** | **H1H2** | **H2H2** | **p-value** |
| No of pigs | |  | 20 | 52 | 36 | - |
| Triglycerides, mg/dL | |  | 33.9±3.2 | 37.8±1.9 | 36.1±2.4 | 0.57 |
| Cholesterol, mg/dL | |  | 119.1±4.2 | 118.8±2.6 | 114.6±3.2 | 0.54 |
| HDL cholesterol, mg/dL | |  | 44.3±1.6 | 44.44±1.0 | 42.8±1.2 | 0.57 |
| LDL cholesterol, mg/dL | |  | 78.6±3.0 | 78.8±1.9 | 75.7±2.2 | 0.56 |
| VLDL, mg/dL | |  | 7.4±0.8 | 7.8±0.6 | 7.0±0.8 | 0.71 |
| Leptin | |  |  |  |  |  |
|  | No of pigs |  | 11 | 39 | 26 | - |
|  | Concentration, ng/mL |  | 44.7±9.5 | 47.2±4.9 | 40.0±6.3 | 0.68 |
| IGF-1 | |  |  |  |  |  |
|  | No of pigs |  | 42 | 138 | 84 | - |
|  | Concentration, ng/mL |  | 75.6±5.0 | 75.8±2.8 | 82.9±3.6 | 0.27 |

^1^HDL: high-density lipoprotein; LDL: low-density lipoprotein; VLDL: very low density lipoprotein; IGF-1: insulin-like growth factor 1.
