# Supplementary material for: A Functional Variant in the Stearoyl-CoA Desaturase Gene Promoter Enhances Fatty Acid Desaturation in Pork
Source: PLoS One. 2014 Jan 20;9(1):e86177. doi: 10.1371/journal.pone.0086177 (PMC3896438; doi:10.1371/journal.pone.0086177)
Supplement: Table S4 — Carcass weight, fat content, and fatty acid composition by SCD diplotype in experimental crossbred pigs. The haplotype H1 showed a favorable effect on 16∶1/16∶0 and 18∶1/18∶0 ratios and no effect on fat content-related traits (carcass weight, lean content, intramuscular fat content, 16∶0+16∶1, and 18∶0+18∶1). Values are expressed as the least square mean (± standard error) for each trait by diplotype. Means lacking a common superscript within trait differ (p<0.05). (DOCX) [file pone.0086177.s005.docx]

**Table S4. Carcass weight, fat content, and fatty acid composition by *SCD* diplotype in experimental crossbred pigs.** The haplotype H1 showed a favorable effect on 16:1/16:0 and 18:1/18:0 ratios and no effect on fat content-related traits (carcass weight, lean content, intramuscular fat content, 16:0+16:1, and 18:0+18:1). Values are expressed as the least square mean (± standard error) for each trait by diplotype. Means lacking a common superscript within trait differ (p<0.05).

|  |  | |  | **Diplotype** | | | |
| --- | --- | --- | --- | --- | --- | --- | --- |
| **Genetic type** | **Trait** | |  | **H1H1** | **H1H2 / H1H3**^a^ | **H2H2** | **p-value** |
| DU-3 × DU-1 | No of pigs | |  | 11 | 28 | 16 | - |
|  | Age at sampling (days) | |  | 206.0 | 206.1 | 205.7 | - |
|  | Carcass weight (kg) | |  | 94.9±2.8 | 95.9±1.7 | 91.5±2.3 | 0.31 |
|  | Backfat depth (mm) | |  | 20.0±1.2 | 19.6±0.7 | 19.1±0.9 | 0.85 |
|  | Lean content (%) | |  | 47.9±1.5 | 48.4±0.9 | 48.8±1.2 | 0.90 |
|  | M. *gluteus medius* | |  |  |  |  |  |
|  |  | IMF (% dry matter) |  | 10.63±1.00 | 12.15±0.62 | 12.11±0.82 | 0.41 |
|  |  | C16:1 (%) |  | 4.01±0.15^a^ | 3.82±0.10^a^ | 3.32±0.13^b^ | 0.001 |
|  |  | C16:1/C16:0 (×100) |  | 17.46±0.72^a^ | 16.81±0.45^a^ | 14.43±0.60^b^ | 0.002 |
|  |  | C16:1 + C16:0 (%) |  | 27.00±0.46 | 26.66±0.29 | 26.37±0.38 | 0.58 |
|  |  | C18:1 (%) |  | 43.28±0.77 | 43.08±0.48 | 42.30±0.63 | 0.53 |
|  |  | C18:1/C18:0 |  | 4.42±0.14^a^ | 4.20±0.08^a^ | 3.71±0.11^b^ | <0.001 |
|  |  | C18:1 + C18:0 (%) |  | 53.10±0.85 | 53.46±0.53 | 53.83±0.70 | 0.80 |
|  |  | MUFA (%) |  | 48.03±0.77 | 47.69±0.48 | 46.39±0.63 | 0.18 |
|  |  | MUFA/SFA |  | 1.39±0.04 | 1.36±0.02 | 1.28±0.03 | 0.07 |
|  |  | MUFA + SFA (%) |  | 82.75±0.82 | 82.90±0.51 | 82.89±0.67 | 0.99 |
| IB-2 × DU-1 | No of pigs | |  | 29 | 25 |  | - |
|  | Age at sampling (days) | |  | 206.6 | 206.6 |  | - |
|  | Carcass weight (kg) | |  | 93.2±1.8 | 91.8±1.9 |  | 0.57 |
|  | Backfat depth (mm) | |  | 24.4±0.3 | 24.4±0.4 |  | 0.96 |
|  | Lean content (%) | |  | 45.5±0.9 | 44.9±0.9 |  | 0.61 |
|  | M. *gluteus medius* | |  |  |  |  |  |
|  |  | IMF (% dry matter) |  | 18.15±0.93 | 17.77±0.97 |  | 0.77 |
|  |  | C16:1 (%) |  | 3.87±0.09^a^ | 3.46±0.09^b^ |  | 0.002 |
|  |  | C16:1/C16:0 (×100) |  | 15.80±0.42^a^ | 14.18±0.43^b^ |  | 0.009 |
|  |  | C16:1 + C16:0 (%) |  | 28.48±0.34 | 27.97±0.35 |  | 0.30 |
|  |  | C18:1 (%) |  | 45.12±0.42 | 44.68±0.44 |  | 0.47 |
|  |  | C18:1/C18:0 |  | 4.14±0.09^a^ | 3.77±0.09^b^ |  | 0.005 |
|  |  | C18:1 + C18:0 (%) |  | 56.11±0.42 | 56.67±0.43 |  | 0.35 |
|  |  | MUFA (%) |  | 49.77±0.44 | 48.90±0.45 |  | 0.17 |
|  |  | MUFA/SFA |  | 1.33±0.03 | 1.28±0.03 |  | 0.15 |
|  |  | MUFA + SFA (%) |  | 87.38±0.37 | 87.31±0.38 |  | 0.89 |
| LW-1 × L-2 | No of pigs | |  | 18 | 25 |  | - |
|  | Age at sampling (days) | |  | 205.5 | 205.5 |  | - |
|  | Carcass weight (kg) | |  | 106.8±1.9 | 102.6±1.7 |  | 0.10 |
|  | Backfat depth (mm) | |  | 18.4±0.8 | 17.7±0.7 |  | 0.46 |
|  | Lean content (%) | |  | 50.7±1.0 | 51.7±0.9 |  | 0.45 |
|  | M. *gluteus medius* | |  |  |  |  |  |
|  |  | IMF (% dry matter) |  | 7.62±0.41 | 7.38±0.37 |  | 0.66 |
|  |  | C16:1 (%) |  | 3.57±0.13 | 3.29±0.12 |  | 0.12 |
|  |  | C16:1/C16:0 (×100) |  | 15.89±0.66 | 14.38±0.59 |  | 0.09 |
|  |  | C16:1 + C16:0 (%) |  | 26.12±0.41 | 26.22±0.37 |  | 0.85 |
|  |  | C18:1 (%) |  | 39.90±0.49 | 39.42±0.44 |  | 0.45 |
|  |  | C18:1/C18:0 |  | 4.10±0.14^a^ | 3.72±0.13^b^ |  | 0.04 |
|  |  | C18:1 + C18:0 (%) |  | 49.85±0.54 | 50.31±0.49 |  | 0.51 |
|  |  | MUFA (%) |  | 44.16±0.55 | 43.40±0.50 |  | 0.30 |
|  |  | MUFA/SFA |  | 1.29±0.03 | 1.23±0.03 |  | 0.11 |
|  |  | MUFA + SFA (%) |  | 78.55±0.69 | 79.02±0.62 |  | 0.60 |

^a^ H1H2 for DU-3 × DU-1 and IB-2 × DU-1; H1H3 for LW-1 × L-2.
