# Supplementary material for: A Functional Variant in the Stearoyl-CoA Desaturase Gene Promoter Enhances Fatty Acid Desaturation in Pork
Source: PLoS One. 2014 Jan 20;9(1):e86177. doi: 10.1371/journal.pone.0086177 (PMC3896438; doi:10.1371/journal.pone.0086177)
Supplement: Table S6 — Sequence of DNA primers used in the characterisation of the porcine SCD gene. A list of the primers used to amplify and sequence seven fragments of the porcine SCD gene encompassing 780 bp of the promoter promoter and the entire coding and 5′ and 3′ non-coding regions (3UTR). The annealing temperature used in the PCR cycling program is also indicated. (DOCX) [file pone.0086177.s007.docx]

**Table S6.** **Sequence of DNA primers used in the characterisation of the porcine *SCD* gene**. A list of the primers used to amplify and sequence seven fragments of the porcine *SCD* gene encompassing 780 bp of the promoter and the entire coding and 5’ and 3’ non-coding regions (3UTR). The annealing temperature used in the PCR cycling program is also indicated.

| **Primer name** | **Sequence 5' 🡪 3'** | **Amplicon size** | **Annealing Tº** |
| --- | --- | --- | --- |
| promoter_F | ACTTCCCTAGTGCCCATCCT | 980 bp | 58ºC |
| promoter_R | GATCACTTTCCCAGGGATGA |  |  |
| cDNA_F | GTCTCATCCCTGGGAAAGTG | 1160 bo | 62ºC |
| cDNA_R | CAGCTGGCTTTCAGAAAAGG |  |  |
| 3UTR_F1 | AAGTATCCAAGGCTGCCATC | 866 bp | 58ºC |
| 3UTR_R1 | CAATTCCGGAAAGAACCTCA |  |  |
| 3UTR_F2 | TGGGGAAGAAGTCTTTCTTGT | 990 bp | 58ºC |
| 3UTR_R2 | GGTTCAGTGACCCTGAGCAT |  |  |
| 3UTR_F3 | TTTCCTGCCGGTTCTATCTC | 945 bp | 60ºC |
| 3UTR_R3 | GAGTAGGTGCTTGGGTCTGG |  |  |
| 3UTR_F4 | ATGGAGGATAAAGGGGTTGG | 648 bp | 60ºC |
| 3UTR_R4 | ACTTGCCCAGGGTCACATAG |  |  |
| 3UTR_F5 | GTCAAGGTTACACGGGTGGT | 742 bp | 58ºC |
| 3UTR_R5 | CAGGACATAGGGTGGCAGAT |  |  |
