# Supplementary material for: A Functional Variant in the Stearoyl-CoA Desaturase Gene Promoter Enhances Fatty Acid Desaturation in Pork
Source: PLoS One. 2014 Jan 20;9(1):e86177. doi: 10.1371/journal.pone.0086177 (PMC3896438; doi:10.1371/journal.pone.0086177)
Supplement: Table S7 — Primers used for genotyping the three single nucleotide polymorphisms (SNPs) in the porcine SCD gene promoter with an allelic discrimination assay. (DOCX) [file pone.0086177.s008.docx]

**Table S7**. **Primers used for genotyping the three single nucleotide polymorphisms (SNPs) in the porcine *SCD* gene promoter with an allelic discrimination assay.**

| **SNP** | **Primer name** | **Sequence 5' --> 3'** | **Final concentration** |
| --- | --- | --- | --- |
| AY487830:*g.2108C>T* | Primer Forward | AGTGTCTGCAGCATCCAGTTTT | 900 nM |
|  | Primer Reverse | GCATGGGCGGGAGAGG | 900 nM |
|  | Probe for G allele | VIC-CCAGCAAGCCCC-NFQ | 200 nM |
|  | Probe for A allele | FAM-CCCAGTAAGCCCC-NFQ | 200 nM |
| AY487830:*g.2228T>C* | Primer Forward | CCCTTCTTGGCAGCGAATAAAA | 900 nM |
|  | Primer Reverse | CAGGCTGGGTATTTAAAGGCTAGAG | 900 nM |
|  | Probe for C allele | VIC-CGACCGTGTCCTGTATT-NFQ | 200 nM |
|  | Probe for T allele | FAM-CGACCGTATCCTGTATT-NFQ | 200 nM |
| AY487830:*g.2281A>G* | Primer Forward | TGCCAGCTCTAGCCTTTAAATACC | 900 nM |
|  | Primer Reverse | CACGTTGGGTCGGTGTCT | 900 nM |
|  | Probe for G allele | VIC-ACCCGCGCACAGCA-NFQ | 200 nM |
|  | Probe for A allele | FAM-AGACCCACGCACAGCA-NFQ | 200 nM |
